# Etiology of Minor Troponin Elevations in Patients with Atrial Fibrillation at Emergency Department–Tropo-AF Study

**DOI:** 10.3390/jcm8111963

**Published:** 2019-11-14

**Authors:** Samuli Jaakkola, Tuomas Paana, Ilpo Nuotio, Tuomas O. Kiviniemi, Jussi-Pekka Pouru, Pekka Porela, Fausto Biancari, K. E. Juhani Airaksinen

**Affiliations:** 1 Heart Center, Turku University Hospital and University of Turku, 20521 Turku, Finland; samuli.jaakkola@tyks.fi (S.J.); tuomas.paana@tyks.fi (T.P.); ilpo.nuotio@tyks.fi (I.N.); tuoski@utu.fi (T.O.K.); jussi-pekka.j.pouru@utu.fi (J.-P.P.); pekka.porela@tyks.fi (P.P.); fausto.biancari@tyks.fi (F.B.); 2 Department of Acute Internal Medicine, Turku University Hospital and University of Turku, 20521 Turku, Finland; 3 Cardiovascular Medicine, Brigham and Women’s Hospital, Harvard Medical School, Boston, MA 02115, USA; 4 Research Unit of Surgery, Anesthesiology and Critical Care, University of Oulu, 90014 Oulu, Finland

**Keywords:** troponin, atrial fibrillation, acute coronary syndrome

## Abstract

Patients with atrial fibrillation (AF) presenting to the emergency department (ED) often have elevated cardiac troponin T (TnT) levels without evidence of type 1 myocardial infarction. We sought to explore the causes and significance of minor TnT elevations in patients with AF at the ED. All patients with AF admitted to the ED of Turku University Hospital between 1 March, 2013 and 11 April, 2016, and at least two TnT measurements, were screened. Overall, 2911 patients with a maximum TnT of 100 ng/L during hospitalization were analyzed. TnT was between 15 and 100 ng/L in 2116 patients. The most common primary discharge diagnoses in this group were AF (18.1%), infection (18.3%), ischemic stroke/transient ischemic attack (10.7%), and heart failure (5.0%). Acute coronary syndrome (ACS) was equally uncommon both in patients with normal TnT and elevated TnT (4.4% vs. 4.5%). Age ≥75 years, low estimated glomerular filtration rate (eGFR), high C-reactive protein (CRP), and hemoglobin <10.0 g/dL, were the most important predictors of elevated TnT. Importantly, TnT elevation was a very frequent (>93%) finding in elderly (≥75 years) AF patients with either low eGFR or high CRP. In conclusion, minor TnT elevations carry limited diagnostic value in elderly AF patients with comorbidities.

## 1. Introduction

Approximately 10–40% of patients with atrial fibrillation (AF) are hospitalized annually and this proportion is expected to rise in the near future [1]. Cardiac troponin measurements have become a part of routine laboratory work-ups in the emergency department (ED) in various clinical scenarios. A common diagnostic challenge with AF patients is the exclusion of type 1 myocardial infarction (MI), referring to infarction caused by plaque rupture, erosion, and/or dissection in coronary arteries, as opposed to type 2 MI, which refers to a mismatch between oxygen supply and demand [2]. The advent of high-sensitive troponin assays enables early detection of acute MI, however, at a cost of false positive diagnoses. Minor troponin elevations are common in a wide variety of medical conditions, such as systemic infection, pulmonary embolism, renal failure, cerebrovascular accident, heart failure, and even after extreme physical stress [3,4,5,6,7,8,9,10]. This multiplicity of causes is problematic in acute care decision-making when the main goal is to detect or rule out type 1 MI as the cause of the ED visit and troponin elevations.

Troponin elevation is common in AF [11,12,13] and patients with AF are a common patient group at the ED. Since little is known about the causes of mild troponin elevations in this scenario, we sought to assess the frequency of various discharge diagnoses in AF patients with minor (15–100 ng/L) cardiac high-sensitivity troponin T (TnT) elevations. Secondly, we sought factors differentiating AF patients with the clinical diagnosis of acute coronary syndrome (ACS) from patients with other causes for minor TnT elevations.

## 2. Materials and Methods

This study reports the main results from the Troponins in Atrial Fibrillation study (The Tropo-AF Study, ClinicalTrials.gov Identifier: NCT03683836), which belongs to a series of study protocols assessing clinical challenges in the treatment of AF [5,14,15,16]. 

The study protocol was approved by the Medical Ethics Committee of the Hospital District of Southwest Finland. Informed consent was not required because of the observational nature of the study. The study complies with the Declaration of Helsinki as revised in 2002.

Initially, database searches were performed to identify patients whose TnT levels were measured between 1 March, 2013 and 11 April, 2016 at the Turku University Hospital laboratory. This initial screening lead to a total of 145,837 individual TnT measurements. Patients with a prior diagnosis of AF (ICD-10 code I48) or AF diagnosed at the index visit, living in the hospital catchment area, with an admission electrocardiography (ECG), and two serial TnT measurements within 8–72 h after admission to the ED, were identified. Thereafter, patients with maximum TnT values < 100 ng/L during the entire hospitalization were selected for the present analysis. Dynamic TnT change was defined as the difference between highest and lowest TnT values recorded during hospitalization, in percentages. Admission ECGs were screened for ST-depression ≥1 mm in two adjacent leads.

The diagnosis of AF (either made before or at the time of the ED visit) was confirmed by 12-lead ECG findings. All individual electronic patient records were reviewed by the investigation group using a standardized protocol to collect specific information on the clinical characteristics of patients, clinical conditions, as well as other clinical and laboratory data of interest from the index hospitalization. Mortality data were retrieved from the national statistical institution, Statistics Finland, and therefore we assumed that no patient was missing at follow-up.

The primary measure of this analysis was an increase of TnT levels between 15 and 100 ng/L during the hospital visit. TnT was analyzed using a commercial high-sensitive assay (Roche Diagnostics GmbH, Mannheim, Germany) at the hospital’s certified laboratory. Determined by the manufacturer, the 99th percentile upper reference limit was 14 ng/L for the assay. Two study groups were formed on the basis of maximum TnT values—patients with normal (<15 ng/L) TnT (*n* = 795) and those with minor (15–100 ng/L) TnT elevations (*n* = 2116).

This article was prepared following the STROBE guidelines [17] for the reporting of observational studies.

### Statistical Analysis

Statistical analyses were performed using SPSS v.25.0 statistical software (IBM Corporation, New York, NY, USA). Continuous variables are reported as mean and standard deviation (SD) if normally distributed, and as median and interquartile range (IQR) if skewed, unless stated otherwise. Missing data were not replaced. Categorical variables are described as counts and percentages. The chi-square test and Fisher’s exact test were used for categorical variables, as appropriate. Logistic regression analysis using the backward Wald method was performed to identify independent predictors of TnT elevations, as well as ACS in patients with elevated TnT. Covariates with *p* < 0.1 in univariate analyses were entered into the regression model. All tests were two-sided and statistical significance was set at 5%. 

Classification and regression tree (CART) analysis was performed to identify independent risk factors for TnT elevations. The classification tree procedure was assessed by cross-validation through 25-fold The minimum number of patients for the parent node was set at 100 and the minimum for the child node was 50. Gini’s method was used to measure impurity, which is the extent to which a node does not represent a homogenous subset of cases. A minimum change in improvement was set at 0.0001. Normalized importance to the model of the included covariates was estimated. Covariates, which were identified as independent risk factors for TnT 15–100 ng/L in logistic regression and with normalized importance >20%, were included in the final CART model. The area under the receiver operating characteristic curve (AUC) was estimated to assess the regression model discrimination. 

## 3. Results

**Figure 1:**
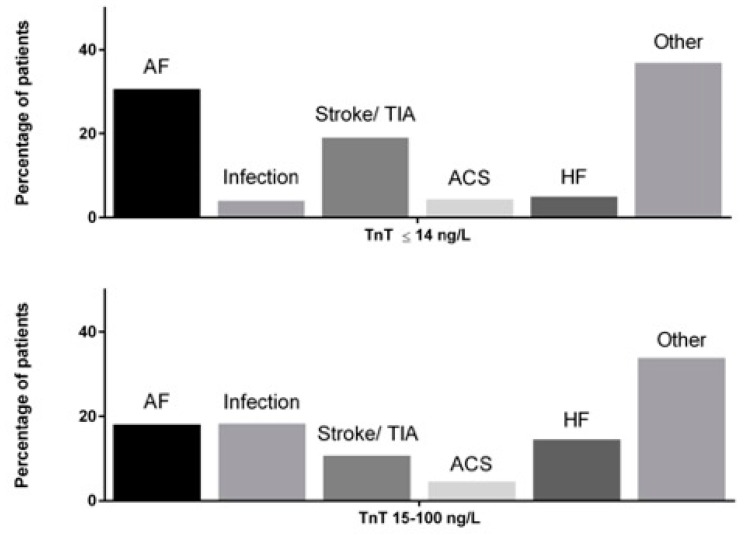
Distribution of different primary discharge diagnoses in patients with normal TnT (**top**) and minor TnT elevations (**bottom**). ACS, acute coronary syndrome; AF, atrial fibrillation; HF, heart failure; and TIA, transient ischemic attack. Linear-by-linear association test: statistically significant (*p* < 0.001) differences in distribution of diagnostic categories between TnT ≤ 14 ng/L and TnT 15–100 ng/L groups.

A total of 2911 AF patients were included in the study and 72.7% (*n* = 2116) of them had minor TnT elevation, ranging from 15 to 100 ng/L. Patient characteristics according to maximum TnT levels are presented in Table 1. The most common symptoms at admission were any kind of chest pain (513 cases, 17.6%), dyspnea (823 cases, 28.3%), and malaise/nausea/dizziness (629 cases, 21.6%). At hospital discharge, the most common primary diagnostic categories were AF (21.6%, *n* = 628), infections (14.4%, *n* = 420), ischemic stroke/transient ischemic attack (13.0%, *n* = 378), decompensated heart failure (11.9%, *n* = 346), and ACS (4.5%, *n* = 130), comprising 65.3% of all hospital admissions (Figure 1 and Table S1). Other frequent primary diagnoses were bone fractures, trauma, intoxications, bleeding, renal insufficiency, inflammatory diseases, and various symptomatic diagnoses. 

Importantly, ACS was an equally uncommon primary discharge diagnosis, both in patients with normal TnT and minor TnT elevations in the ED (4.4%, *n* = 35 vs. 4.5%, *n* = 95, respectively). AF was the most common (30.7%, *n* = 244) primary discharge diagnosis of the index hospital visit in patients with normal TnT, whereas severe infections (18.3%, *n* = 388) and AF (18.1%, *n* = 384) were the most frequent diagnoses at hospital discharge in patients with minor TnT elevations (Figure 1). 

Chest pain was reported by 27.0% (*n* = 215) of patients with normal TnT, while only 14.1% (*n* = 298) of those with minor TnT elevations reported chest pain. Among patients with ACS, nearly two-thirds (65.4%, *n* = 85) experienced chest pain, but only 12.8% (*n* = 38) of patients with chest pain and minor TnT rises were diagnosed with ACS. ST-depressions were observed in 10.7% of patients with ACS and 13.2% of those with other primary diagnoses. A coronary angiogram was performed in 3.0% (*n* = 87) of all study patients and in 24.6% (*n* = 32) of patients with the final diagnosis of ACS. Coronary angiography revealed significant coronary artery disease (>50% stenosis) in 31 (96.9%) ACS patients, but resulted in percutaneous coronary intervention in 18 (20.7%) patients, including two patients with normal TnT values. Patients were selected for coronary angiography based on clinical evaluation by the attending physician. 

**Figure 2:**
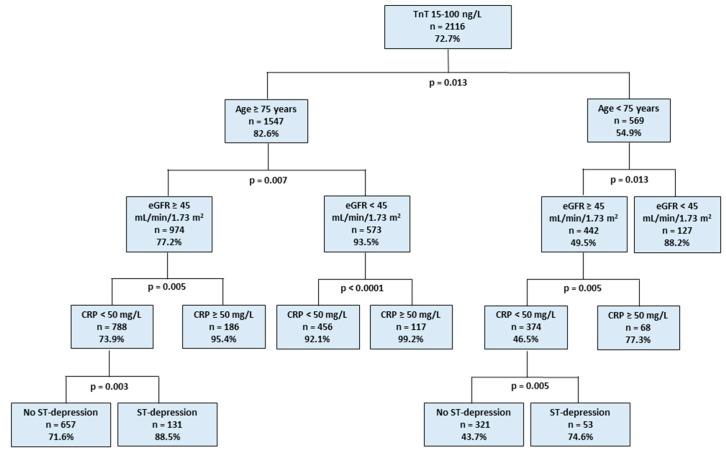
Classification and regression tree analysis showing the independent importance of covariates predicting troponin T elevations in patients with atrial fibrillation. %: percentage of patients with TnT 15–100 ng/L among patients with covariates indicated in each box. CRP, C-reactive protein; eGFR, estimated glomerular filtration rate (calculated using the Chronic Kidney Disease Epidemiology Collaboration equation); TnT, high-sensitivity cardiac troponin T. ST-depression: ≥1 mm ST-depression in two adjacent leads.; eGFR, estimated glomerular filtration rate; CRP, C-reactive protein.

The strongest independent predictors of minor TnT elevation in the whole patient cohort were age ≥ 75 years, estimated glomerular filtration rate (eGFR) < 45 mL/min/1.73 m^2^, C-reactive protein (CRP) ≥ 50 mg/L, and hemoglobin < 10.0 g/dL (Table 2). CART analysis showed that nearly all (>93%) patients ≥ 75 years of age with either high CRP or low eGFR had elevated TnT concentrations (Figure 2). Age ≥ 75 years (normalized importance, 100%), eGFR < 45 mL/min/1.73 m^2^ (normalized importance, 57.6%), CRP ≥ 50 mg/L (normalized importance, 30.5%), and ST depression ≥1 mm (normalized importance, 21.0%), were included in the final CART model. The AUC of this CART model was 0.763 (95% CI, 0.745–0.782). A CART model not including patients with a diagnosis of ACS confirmed the findings of the overall cohort (AUC 0.763, 95% CI, 0.744–0.782).

In patients with elevated TnT, chest pain (OR 8.29, 95% CI, 5.20–13.2), previous coronary artery disease (OR 2.33, 95% CI, 1.42–3.81), and low eGFR (OR 1.65, 95% CI, 1.04–2.62) were the independent predictors for the final diagnosis of ACS (Table 3), but only 24.1% of minor TnT elevations in patients with previous coronary artery disease and chest pain were considered to be caused by ACS. A change of at least 100% in absolute TnT value was observed in 12 (12.6%) patients with ACS and in 146 (7.2%) without ACS, *p* = 0.068. 

The 30-day mortality was significantly higher in the TnT elevation group compared to the normal TnT group, both in the whole study cohort and in patients with ACS (8.9% vs. 0.9% and 8.4% vs. 2.9%, *p* < 0.001 for both). One (0.05%) patient with elevated TnT and no ACS diagnosis during the index hospitalization was treated for ACS during the 30-day follow-up.

## 4. Discussion

Our study demonstrated that minor TnT elevation is a common finding in AF patients presenting at the ED with a wide variety of acute symptoms. Strikingly, ACS or type 1 MI was considered a rare cause of minor TnT elevations, comprising less than one out of twenty patients. Increasing age and multiple co-morbidities were often associated with minor TnT elevations; severe infections, cerebrovascular events, AFs, and decompensated heart failure were the most common final diagnoses at discharge. Most patients with minor TnT elevations were admitted to hospital and minor TnT elevation was a powerful predictor of short-term mortality regardless of the final diagnosis. Importantly, minor TnT elevation by itself did not predict a new ED admission due to ACS within 30 days.

The use of high-sensitivity troponin assays enables safe and robust early exclusion of acute MI in the ED [18,19]. High-sensitivity assays also allow the use of lower thresholds for the diagnosis of type 1 MI. The use of this improved sensitivity, however, more than doubles the number of patients with type 2 MI or myocardial injury, but does not seem to improve the prognosis of this new patient group with minor troponin elevations [20]. The present results show that the recommended low rule-out threshold for exclusion of type 1 MI is problematic in clinical practice if applied to patients in whom the degree of MI suspicion is low and there are other factors that might contribute to troponin concentration. 

At present, cardiac troponin testing is frequently used in the ED in the absence of symptoms suggestive of ACS [21]. In line with earlier reports [22], the vast majority of minor TnT elevations in this scenario reflected myocardial injury or type 2 MI rather than type 1 MI. Earlier reports have shown that if troponin testing is done in all ED patients without selection, elevated cardiac troponin concentrations are frequent, occurring in one in every eight patients [23], and the prevalence of type 1 MI is very low (1.6%) [24]. Furthermore, high-sensitivity troponin is elevated in over 12% of ED patients without any clinical suspicion of ACS [23,24].

Minor TnT elevation was a common finding in AF patients presenting to the ED with a variety of acute symptoms. Older age was a strong predictor of elevated TnT and the majority of AF patients older than 75 years had abnormal TnT. If combined with other strong predictors, i.e., either high CRP or impaired renal function, almost all patients had minor TnT elevations irrespective of their final diagnosis (Figure 2), and minor TnT elevations had no diagnostic value in these patient groups. Taking this into consideration, an age-adjusted TnT threshold might prove useful in reducing the rate of unjustified diagnostic efforts to exclude ACS in patients with non-coronary-type symptoms.

As expected, previous history of coronary artery disease and chest pain in the ED were the strongest predictors of ACS in AF patients with minor TnT elevations. Even in this clinical scenario, TnT elevations were considered to be caused by ACS in only a quarter of cases. Our analysis included only patients with serial TnT testing, and it was supposed that dynamic elevation of TnT would increase the specificity of TnT elevations [25,26,27], although contradictory data has also been published [28]. Disappointingly, dynamic TnT changes (rises or decreases) did not have additional predictive power in the diagnosis of ACS. This observation was, however, not that unexpected since most of the patients with TnT elevations were admitted to hospital because of acute severe illnesses known to cause temporary TnT elevation [29,30,31,32,33,34].

Patients with type 2 MI or myocardial injury have not been shown to benefit from aggressive antithrombotic treatments or invasive investigation and treatment, unlike patients with type 1 MI. Thus, indiscriminate troponin testing in patients without signs or symptoms consistent with ACS is likely to increase diagnostic uncertainty, with no improvements in patient outcomes. Minor troponin elevations may lead to inappropriate diagnostic calibration in the use of coronary angiography, inappropriate resource use, and lengthened hospital admissions. In the present study, only every fifth coronary angiography revealed coronary changes leading to coronary intervention. 

A slew of mechanisms of myocardial injury resulting in troponin release, besides myocardial ischemia, have been characterized. Those mechanisms include cell injury, apoptosis, myocardial strain, inflammation, and/or those decreasing troponin clearance, such as acute or chronic renal impairment [35,36]. The causes underlying non-coronary disease-related minor troponin elevation in AF are not fully clear; even minor increases in troponin levels are associated with increased mortality risks in several settings [23,37]. 

Some limitations of our study should be acknowledged. This is an observational study, including comprehensive data collected from AF patients suffering from a variety of acute symptoms and having at least two TnT measurements during the hospital visit. Patients were treated by a heterogeneous group of emergency medicine physicians with no strict institutional protocol for when to test for TnT, but all measurements require a physician order. The final diagnoses were based on real-life adjudication by the treating physicians in the ED and at wards, with all known uncertainties and potential misjudgments. Our intent was to identify AF patients with ACS among patients with minor TnT elevations in the ED, since the specific diagnosis of type 1 MI is problematic in this study setting. Against our preconceptions, diagnostic coronary angiography was seldom used in the diagnostic work-up, increasing the uncertainty and potential missing of ACS cases. It is, however, noteworthy that ACS was extremely uncommon (0.05%) during the following month in the patients with elevated TnT during the index ED visit. 

## 5. Conclusions

Minor TnT elevations are common in patients with AF presenting at the ED with a variety of symptoms and most of them reflect myocardial injury of type 2 MI. ACS is seldom a cause of minor TnT elevations even in patients with chest pain. Minor TnT elevations do not provide diagnostic value in older patients with multiple co-morbidities. Troponin testing should not be used for unselected ACS screening in ED patients with non-specific symptoms. Clinicians should be aware of the other multiple contributory factors when interpreting elevated cardiac troponin concentrations in their practice.

## Figures and Tables

**Table 1:** Patient characteristics according to troponin T levels at hospital admission.

Characteristic	TnT ≤ 14 ng/L (*n* = 795)	TnT 15–100 ng/L (*n* = 2116)	
	No. (%)	No. (%)	*p*
Age, median (IQR), y	72.0 (14)	81.0 (12)	<0.001
Women	431 (54.3)	1026 (48.5)	0.005
CHA_2_DS_2_-VASc, mean (SD)	3.30 (1.85)	4.11 (1.68)	<0.001
Heart failure	86 (10.8)	524 (24.8)	<0.001
Hypertension	505 (63.5)	1485 (70.2)	0.001
Diabetes mellitus	155 (19.5)	590 (27.9)	<0.001
Prior stroke	119 (15.0)	346 (16.4)	0.39
Prior myocardial infarction	99 (12.5)	350 (16.5)	0.007
Hypercholesterolemia	340 (42.8)	811 (38.3)	0.03
Coronary artery disease	186 (23.4)	683 (32.3)	<0.001
Heart rate at admission, median (IQR), bpm	81 (66–102)	87 (72–109)	<0.001
Systolic blood pressure, median (IQR), mm Hg *	144 (126–163)	140 (122–160)	0.002
ST depression in admission ECG *^,†^	42 (5.8)	305 (15.9)	<0.001
Laboratory tests at admission			
TnT, median (IQR), ng/L	9 (6–11)	30 (21–48)	<0.001
Hemoglobin, median (IQR), g/dL	13.8 (12.8–14.7)	12.8 (11.5–14.2)	<0.001
Creatinine, median (IQR), μmol/L	79.6 (70.7–97.2)	97.2 (79.6–124)	<0.001
eGFR, median (IQR), mL/min/1.73 m^2^	71.3 (58.8–83.8)	55.1 (40.0–72.3)	<0.001
ProBNP, median (IQR), ng/L *	998 (454–2335)	3370 (1580–6355)	<0.001
CRP, median (IQR), mg/L	3 (2–7)	10 (3–37)	<0.001

CHA_2_DS_2_-VASc, 1 point each for congestive heart failure, hypertension, diabetes mellitus, vascular disease, age 65–74 years, and female sex, and 2 points for prior stroke, transient ischemic attack, or thromboembolism, and age ≥ 75 years; CRP, C-reactive protein; eGFR, estimated glomerular filtration rate (calculated using the Chronic Kidney Disease Epidemiology Collaboration equation); TnT, high-sensitivity cardiac troponin T, ProBNP, pro-brain natriuretic peptide. *, The number of patients with missing data for CRP was *n* = 261 (9.0%), for ST-depression *n* = 265 (9.1%), for systolic blood pressure *n* = 352 (12.1%), and for proBNP *n* = 1507 (51.8%). ^†^, ≥1 mm ST-depression in two adjacent leads; IQR, interquartile range; SD, standard deviation.

**Table 2:** Predictors of troponin T elevation in patients with atrial fibrillation.

Univariable	Odds Ratio (95% CI)	*p*	Multivariable	Odds Ratio (95% CI)	*p*
Age ≥ 75 years	3.89 (3.28–4.62)	<0.001	Age ≥ 75 years	4.77 (3.77–6.04)	<0.001
Male gender	1.26 (1.07–1.49)	0.005	Male gender	2.54 (2.01–3.21)	<0.001
Heart failure	2.71 (2.13–3.47)	<0.001	Chest pain	0.58 (0.44–0.76)	<0.001
Hypertension	1.35 (1.14–1.61)	0.001	ST depression in admission ECG *	2.71 (1.85–3.97)	<0.001
Diabetes mellitus	1.60 (1.31–1.95)	<0.001	Ventricular rate ≥ 100 bpm at admission	1.71 (1.36–2.17)	<0.001
Prior stroke or TIA	0.98 (0.80–1.20)	0.83	eGFR < 45 mL/min/1.73 m^2^	5.06 (3.58–7.14)	<0.001
Hypercholesterolemia	0.83 (0.71–0.98)	0.03	CRP ≥ 50 mg/L	3.79 (2.49–5.77)	<0.001
Coronary artery disease	1.56 (1.29–1.89)	<0.001	Hemoglobin < 10.0 g/dL	4.95 (2.33–10.55)	<0.001
Active malignancy	1.30 (0.85–1.98)	0.23			
Chest pain	0.44 (0.36–0.54)	<0.001			
ST depression in admission ECG *^,†^	3.06 (2.19–4.27)	<0.001			
Ventricular rate ≥ 100 bpm at admission	1.37 (1.14–1.64)	0.001			
AF at admission	1.79 (1.51–2.12)	<0.001			
eGFR < 45 mL/min/1.73 m^2^	6.40 (4.82–8.51)	<0.001			
CRP ≥ 50 mg/L ^†^	5.04 (3.46–7.35)	<0.001			
Hemoglobin < 10.0 g/dL	6.19 (3.34–11.4)	<0.001			

AF, atrial fibrillation; CI, confidence interval; CRP, C-reactive protein; ECG, electrocardiography. eGFR, estimated glomerular filtration rate (calculated using the Chronic Kidney Disease Epidemiology Collaboration equation); TIA, transient ischemic attack. * ≥1 mm ST-depression in two adjacent leads. ^†^ The number of patients with missing data for CRP was *n* = 261 (9.0%) and for ST-depression *n* = 265 (9.1%)

**Table 3:** Predictors of acute coronary syndrome among patients with atrial fibrillation and elevated cardiac troponin T.

Univariable Predictors	Odds Ratio (95% CI)	*p*	Multivariable Predictors	Odds Ratio (95% CI)	*p*
Age ≥ 75 years	0.88 (0.56–1.37)	0.561	Chest pain	8.29 (5.20–13.2)	<0.001
Male gender	1.73 (1.13–2.66)	0.012	Coronary artery disease	2.33 (1.42–3.81)	0.001
Hypertension	1.43 (0.88–2.33)	0.148	eGFR < 45 mL/min/1.73 m^2^ *	1.65 (1.04–2.63)	0.035
Diabetes mellitus	1.69 (1.11–2.59)	0.015			
Hypercholesterolemia	2.52 (1.66–3.84)	<0.001			
Prior myocardial infarction	3.83 (2.50–5.87)	<0.001			
Prior stroke or TIA	0.67 (0.37–1.21)	0.186			
Coronary artery disease	4.68 (3.01–7.26)	<0.001			
Heart failure	0.91 (0.56–1.48)	0.711			
Chest pain	11.1 (7.19–17.1)	<0.001			
eGFR < 45 mL/min/1.73 m^2^	1.57 (1.03–2.37)	0.035			
CRP ≥ 50 mg/L	0.38 (0.18–0.80)	0.009			
Troponin T, ≥100% change	1.86 (0.99–3.48)	0.054			
Hemoglobin < 100 g/L	1.06 (0.51–2.23)	0.873			
ST depression in ECG *	0.73 (0.37–1.43)	0.355			

CI, confidence interval; CRP, C-reactive protein; ECG, electrocardiogram; eGFR, estimated glomerular filtration rate (calculated using the Chronic Kidney Disease Epidemiology Collaboration equation); TIA, transient ischemic attack. *, ≥1 mm ST-depression in two adjacent leads.

## Supplementary Materials

The following are available online at https://www.mdpi.com/2077-0383/8/11/1963/s1, Table S1: Patient Characteristics According to Primary Diagnosis at Hospital Discharge.
