# Supplementary material for: Etiology of Minor Troponin Elevations in Patients with Atrial Fibrillation at Emergency Department–Tropo-AF Study
Source: J Clin Med. 2019 Nov 14;8(11):1963. doi: 10.3390/jcm8111963 (PMC6912339; doi:10.3390/jcm8111963)
Supplement: Supplementary file 1 [file jcm-08-01963-s001.pdf]

Supplementary Materials:

Table S1. Patient Characteristics According to Primary Diagnosis at Hospital Discharge.

Characteristic	Atrial fibrillation (n=628) No. (%)	Infection (n=420) No. (%)	Stroke/TIA (n=378) No. (%)	ACS (n=130) No. (%)	Heart Failure (n=346) No. (%)	Other (n=1009) No. (%)
Age, median (IQR), y	75 (66-82)	82 (74-87)	78 (70-84)	78 (71-84)	82 (75-87)	79 (71-85)
Women	335 (53.3)	210 (50.0)	200 (52.9)	47 (36.2)	173 (50.0)	493 (48.9)
CHA_2DS_2-VASc, mean (SD)	3.2 (1.7)	4.2(1.7)	4.0 (1.8)	4.2 (1.5)	4.6 (1.6)	3.9 (1.8)
Congestive heart failure	57 (9.1)	117 (27.9)	48 (12.7)	24 (18.5)	163 (47.1)	201 (19.9)
Hypertension	398 (63.4)	293 (69.8)	272 (72.0)	95 (73.1)	249 (72.0)	683 (67.7)
Diabetes	116 (18.5)	117 (27.9)	80 (21.2)	45 (34.6)	128 (37.0)	259 (25.7)
Prior stroke	58 (9.2)	79 (18.8)	93 (24.6)	13 (10.0)	60 (17.3)	162 (16.1)
Prior myocardial infarction	83 (13.2)	65 (15.5)	46 (12.2)	48 (36.9)	73 (21.1)	134 (13.3)
Hypercholesterolemia	245 (39.0)	139 (33.1)	147 (38.9)	75 (57.7)	148 (42.8)	397 (39.3)
Coronary artery disease	147 (23.4)	127 (30.2)	87 (23.0)	88 (67.7)	131 (37.9)	289 (28.6)
Prior coronary bypass surgery	39 (6.2)	33 (7.9)	20 (5.3)	30 (23.1)	41 (11.8)	82 (8.1)
Prior percutaneous coronary intervention	51 (8.1)	31 (7.4)	26 (6.9)	36 (27.7)	44 (12.7)	82 (8.1)
Ventricular rate at admission, median (IQR), bpm	113 (88-133)	91 (76-109)	75 (64-88)	81(68-92)	86 (73-101)	79 (65-79)
Systolic blood pressure, median (IQR), mm Hg*	135 (119-151)	134 (113-155)	160 (141-178)	140 (125-157)	143 (121-164)	123 (123-161)
TnT at admission, median (IQR), ng/L	17 (10-28)	34 (23-52)	17 (9-28)	26 (12-53)	33 (20-50)	23 (12-40)

Abbreviations: CHA2DS2-VASc 1 point each for congestive heart failure, hypertension, diabetes mellitus, vascular disease, age 65-74 years, and female sex, and 2 points for prior stroke or transient ischemic attack or thromboembolism, and age ≥ 75 years; TnT, high-sensitivity cardiac troponin T; ProBNP, pro-brain natriuretic peptide * Data missing in 352 patients (12.1%).
